# Selective hydrogenation of diphenylacetylene using NiCo nanoparticles supported on mesoporous carbon as catalyst

**DOI:** 10.55730/1300-0527.3359

**Published:** 2021-11-30

**Authors:** Alyaa S. SHIDDIQAH, Iman ABDULLAH, Yuni K. KRISNANDI

**Affiliations:** 1 Department of Chemistry, Faculty of Mathematics and Natural Sciences, University of Indonesia, Depok, Indonesia; 2 Solid Inorganic Framework Laboratory, Department of Chemistry, Faculty of Mathematics and Natural Sciences, University of Indonesia, Depok, Indonesia

**Keywords:** mesoporous carbon, NiCo catalyst, diphenylacetylene, selective hydrogenation

## Abstract

Hydrogenation of alkynes to alkenes is an important procedure in the synthesis of organic compounds. In this study, selective hydrogenation was carried out on diphenylacetylene as a model of alkyne compounds using NaBH_4_ as a hydrogen source and NiCo bimetallic nanoparticles supported on mesoporous carbon (NiCo/MC) as a catalyst. The mesoporous carbon was prepared using the soft-templated method from phloroglucinol and formaldehyde as precursors while the NiCo/MC catalyst was synthesized using a wet impregnation method. Based on surface area analysis, it was found that the pore diameters of MC, Ni/MC, and NiCo/MC were 12.8 nm, 13.4 nm, and 12.7 nm respectively, which indicated the mesoporous size of the materials. TEM analysis also confirmed the formation of nanoparticles on mesoporous carbon with the average size similar to the pore structure of the support, thus indicating the incorporation of the metals on the support. The hydrogenation reaction of diphenylacetylene was carried out with variations in reaction time and temperature. GCMS analysis of the products showed that the optimum conditions were obtained over NiCo/MC catalyst at 50 °C for 4 h with a diphenylacetylene conversion of 71.5% and a selectivity of 87.1% for the formation of *cis*-stilbene.

## 1. Introduction

The reduction and isomerization of alkyne compounds are important in organic synthesis [1]. In general, frequently used methods to reduce alkyne are direct hydrogenation with molecular hydrogen and transfer hydrogenation. The advantages of transfer hydrogenation are that it does not use flammable compressed H_2_ gas, hydrogen donors are easier to handle, easy for the recycling of side-products, and more inexpensive [2]. Therefore, solid compounds that can release hydrogen are used, for example, sodium borohydride or NaBH_4_ [3]. Although hydrogenation with NaBH_4_ is slow, the process can be accelerated in the presence of a catalyst. Examples of reported methods related to alkynes hydrogenation using NaBH_4_ as reducing agents using homogeneous catalysts are hydrogenation of styrene to ethyl-benzene using bimetallic Ni(II) complex, and hydrogenation of alkynes using colloidal nickel nanoparticles [2, 4, 5].

Nickel as a catalyst for transfer hydrogenation of alkyne compounds has several advantages, namely, it is relatively inexpensive, abundant, and environmentally friendly [2]. Nickel itself has long been known to have a good affinity for hydrogen and thus, serves as a good catalyst in the hydrogenation of unsaturated bonds. However, nickel alone sometimes is not sufficient to give high catalytic activity and selectivity, especially when used in the form of heterogeneous catalysts. On the other hand, the catalytic property of nickel can be enhanced by modification with other metals in the form of bimetallic Ni-M. Various nickel based-bimetallic catalysts have been reported to be successfully synthesized for various applications such as Ni-Fe as a catalyst for hydrogen generation from sodium borohydride [6], Ni-Sn as a catalyst for hydrogenation of 2-methylfuran [7], Ni-Cu as a catalyst in nitrophenol reduction [8], Ni-Zn as a catalyst in carboxylation with CO_2_ [9], Ni-Ga as a catalyst in the reduction of CO_2_ to methanol [10], Ni-Ag as a catalyst in acetylene hydrogenation [11], and some Ni-noble metals for CO_2_ reforming of methane [12]. Recently, Yang et al [13] reported the use of bimetallic Ni-M (M = Zn, Ga, Cu, and Fe)/AlSBA-15 as a catalyst in the semihydrogenation of phenylacetylene. The results showed that NiZn_3_/AlSBA-15 gave the highest styrene selectivity, 90.3%. Bimetallic NiCo on TiO_2_ has also been reported as a promising catalyst for the dehydrogenation of hydrazine hydrate [14].

Meanwhile, mesoporous carbon as catalyst support has attracted great interest because it has a large surface area and pore distribution. Mesoporous carbon is also nontoxic, noncorrosive, environmentally friendly, and can be used repeatedly [15]. Previously, we have reported the utilization of mesoporous carbon as solid support for various nickel species [16–18], bimetallic nickel-zinc [9], and copper [19]. In this report, we present the use of bimetallic NiCo supported on mesoporous carbon (NiCo/MC) as a catalyst in selective hydrogenation reaction of diphenylacetylene to *cis*-stilbene with NaBH_4_ as a hydrogen source/reducing agent. The hydrogenation reactions were carried out at variations in time and temperature to determine the optimum conditions. The catalytic activity of NiCo/MC was also compared to the common monometallic Ni/MC for the same reaction to investigate the role of the second metal (Co) on the catalytic reaction.

## 2. Materials and methods

### 2.1. Materials

The materials used were phloroglucinol 99% (Sigma Aldrich), pluronic F127 (Sigma Aldrich), ethanol 97% (Merck), HCl 37% (J.T Baker), formaldehyde 37% (Merck), nickel (II) nitrate (Merck), cobalt (II) nitrate (Merck), diphenylacetylene 98% (Sigma Aldrich), sodium borohydride powder ≥ 98% (Sigma Aldrich), methanol 99% (Merck), cis-stilbene 96% (Sigma Aldrich), N_2_ gas, H_2_ gas, and deionized water.

### 2.2. Synthesis of catalysts

#### 2.2.1. Synthesis of mesoporous carbon

The mesoporous carbon (MC) was prepared via a soft template method using a standard reported procedure with modifications [20]. The synthesis was started by dissolving 1.25 g phloroglucinol and 1.25 g pluronic F-127 in 10 mL of 9:10 (w/w) ethanol-water mixture. Then 0.08 mL of 37% HCl was added, followed by the addition of 1.25 mL of 37% formaldehyde. The solution was stirred for 2 h until two phases were formed. The bottom layer was taken, then stirred for 12 h, and left for 24 h at room temperature until a monolith was formed. The monolith was put into an autoclave and heated at 100 °C in an oven for 24 h. The solid was then carbonized in a tubular furnace with N_2_ gas flow with a heating rate of 5 °C/min at a temperature range of 100–850 °C. The carbonization temperature was maintained at 850 °C for 2 h.

#### 2.2.2. Synthesis of Ni/MC and NiCo/MC

The synthesis of Ni/MC was performed by wet impregnation method. First, a 0.085 M nickel nitrate solution was prepared by dissolving an appropriate amount of nickel nitrate powder in a 1:1 (w/w) water-ethanol mixture. Then, 0.5 g MC powder was dispersed into the solution by sonication for 10 min, followed by stirring for 48 h at room temperature, dried in an oven for 6 h at 100 °C and finally reduced by H_2_ gas flow at 400 °C for 4 h. The synthesis of NiCo/MC was prepared by the same method as Ni/MC using an equimolar concentration of nickel and cobalt nitrate as precursors. The synthesized catalysts were then characterized using FTIR, XRD, SEM-EDX, TEM, and SAA-BET.

### 2.3. Hydrogenation of diphenylacetylene

Hydrogenation of diphenylacetylene was conducted by dissolving 0.1782 g diphenylacetylene (1 mmol) in 8 mL of methanol in a round flask. After stirring for 10 min, 0.0757 g NaBH_4_ (2 equiv) and 0.0450 g Ni/MC or NiCo/MC was added. The mixture was stirred, and the reaction was carried out for 2, 4, and 6 h, with temperature variations of 30 °C and 50 °C. The reaction products were characterized using GC-MS.

## 3. Results and discussion

### Characterization of catalysts

**Figure 1:**
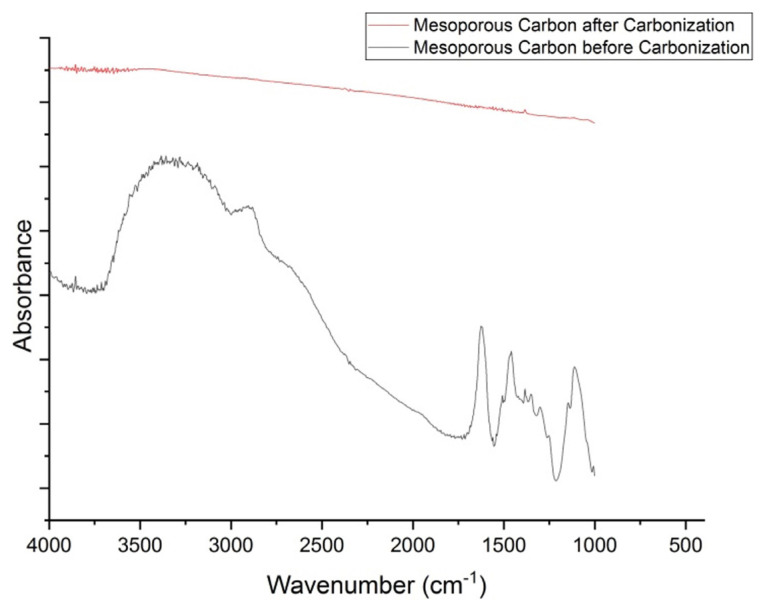
FTIR spectra of mesoporous carbon before and after carbonization.

Mesoporous carbon was characterized by FTIR to determine the success of carbonization. Figure 1 shows the difference spectrum between mesoporous carbon before and after carbonization. Before the carbonization, it shows the absorption peak around 3200–3600 cm^−1^ which is the peak of OH stretching vibration, 2850–3000 cm^−1^ which is the peak of CH stretching vibration, and 1600–1700 cm^−1^ which is the vibrational peak of the C = C stretching. The spectrum after carbonization indicates the absence of the absorption peaks, showing that the carbonization process has succeeded in functional groups decomposition of the precursors.

**Figure 2:**
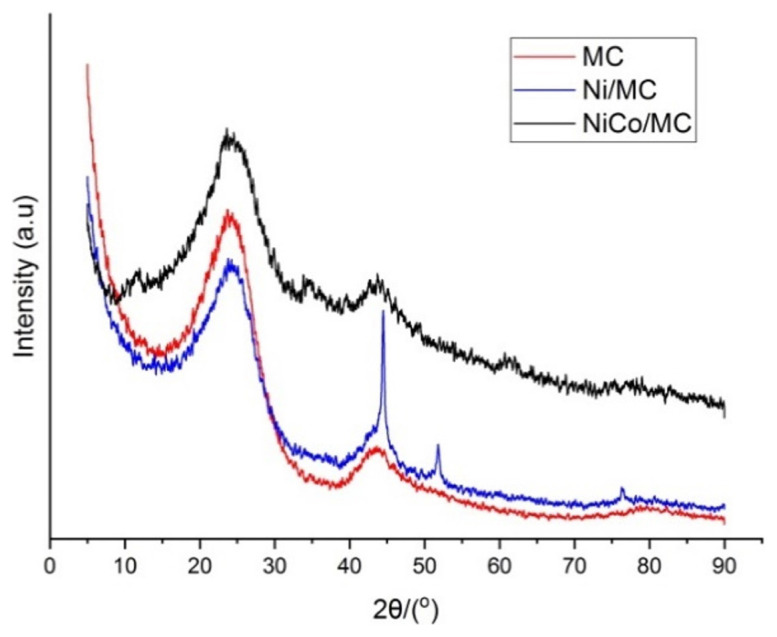
X-Ray diffraction patterns of synthesized mesoporous carbon, Ni/MC, and NiCo/MC.

Figure 2 shows the X-Ray diffraction pattern of mesoporous carbon, Ni/MC, and NiCo/MC. X-Ray diffraction pattern of MC shows two peaks at 2θ of 22.9° and 43.1° which are the typical peaks for carbon material according to JCPDS index No.75-1621 [21]. The peaks also indicate that the synthesized mesoporous carbon has an amorphous structure. The diffraction pattern of Ni/MC (blue line) shows three additional peaks detected at 44.46°, 51.88°, and 76.38° in addition to the baseline peaks of MC. The additional peaks show the presence of nickel metal in the form of cubic crystals according to JCPDS No. 04-0850 [22]. The diffraction pattern of NiCo/MC shows peaks at 23.42°, 34.76°, 43.67°, 61.54°, 75.37°, and 77.00°. According to JCPDS No.15-0806, cobalt metal has three distinctive peaks of diffraction at 44.1°, 51.4°, and 75.9° while according to JCPDS No. 75-1621, cobalt oxide shows diffraction peaks at 36.5, 42.4, 61.5, 73.5, and 77.5. Therefore, the NiCo/MC XRD pattern indicates that in addition to the formation of nickel and cobalt metals, there is also the formation of cobalt oxide on the mesoporous carbon surface.

**Figure 3:**
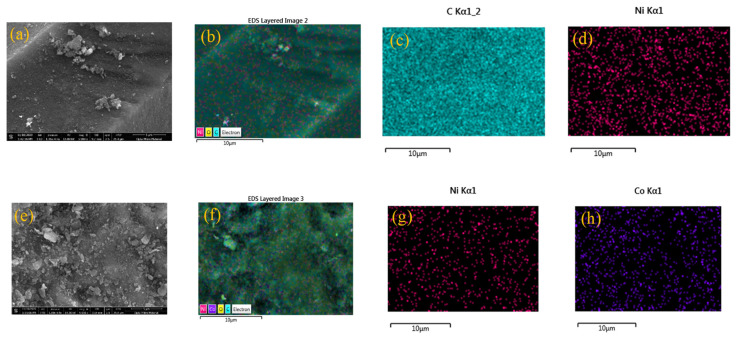
(a) SEM images of Ni/MC; (b) combined elemental mapping image of Ni/MC; (c) carbon mapping image of Ni/MC; (d) nickel mapping image of Ni/MC; (e) SEM images of NiCo/MC; (f) combined elemental mapping image of NiCo/MC; (g) nickel mapping image of NiCo/MC; (h) cobalt mapping image of NiCo/MC.

SEM images of Ni/MC and NiCo/MC are shown in Figure 3a–3h. The figures show the presence of fine grains on the surface of materials. EDX mapping of Ni/MC reveals that the nickel metal is distributed evenly on MC (Figure 3d). The same result was obtained in NiCo/MC, both nickel and cobalt metal have been deposited on MC evenly (Figure 3g and 3h). The EDX analysis showed a Ni loading in Ni/MC at 3.7% by weight. Meanwhile the Ni and Co content in NiCo/MC is 1.1% and 1.2%, respectively.

**Figure 4:**
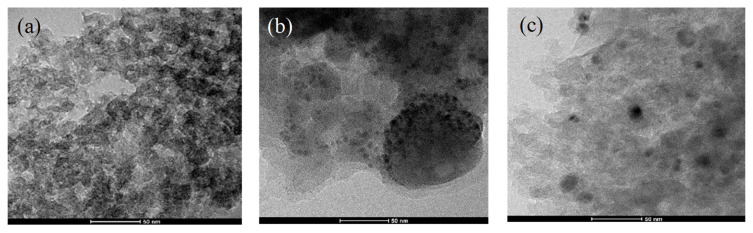
TEM images of (a) mesoporous carbon (b) Ni/MC, and (c) NiCo/MC.

**Figure 5:**
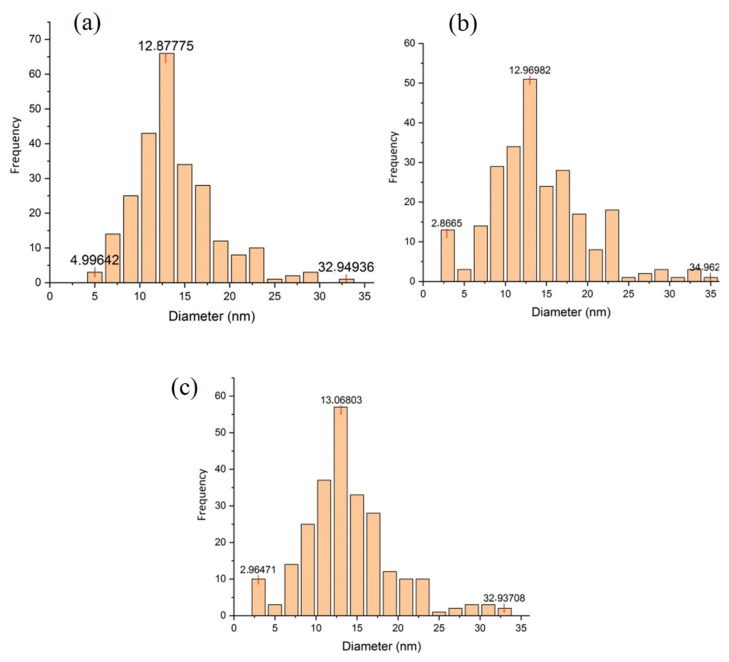
Histogram of pore size distribution of (a) mesoporous carbon, particle diameter of (b) Ni/MC, and (c) NiCo/MC.

Characterization using transmission electron microscopy (TEM) was used to see the morphology and particle distribution at higher magnification and resolution. Based on Figure 4a, the mesoporous carbon has a spherical pore structure that forms a wormhole-like mesoporous channel with a random pore orientation. TEM images of Ni/MC and NiCo/MC (Figure 4b and Figure c) show that nickel and bimetallic nickel-cobalt metals are evenly distributed on mesoporous carbon. Apart from the metal appearance on mesoporous carbon, the porous structure of MC can still be observed around the black metal spot, which indicates that the carbon matrix remains intact after impregnation with metal [23]. ImageJ software was used to determine the diameter and number of pores in mesoporous carbon, as well as the diameter of Ni and NiCo nanoparticles on Ni/MC and NiCo/MC. The results are presented in the following histogram (Figure 5). Before impregnation, MC had a pore diameter of 4 nm to 33 nm with a dominant pore size of 12.9 nm (Figure 5a). After impregnation, Ni particles formed in MC have a particle size range of 2.9 to 35 nm, with the dominant particle diameter being 13 nm (Figure 5b). The same result was observed in the bimetallic NiCo (Figure 5c). These results indicate that in general, the size of the metal particles formed corresponds to the available MC pores, which is in accordance with the role of MC as metal support.

**Figure 6:**
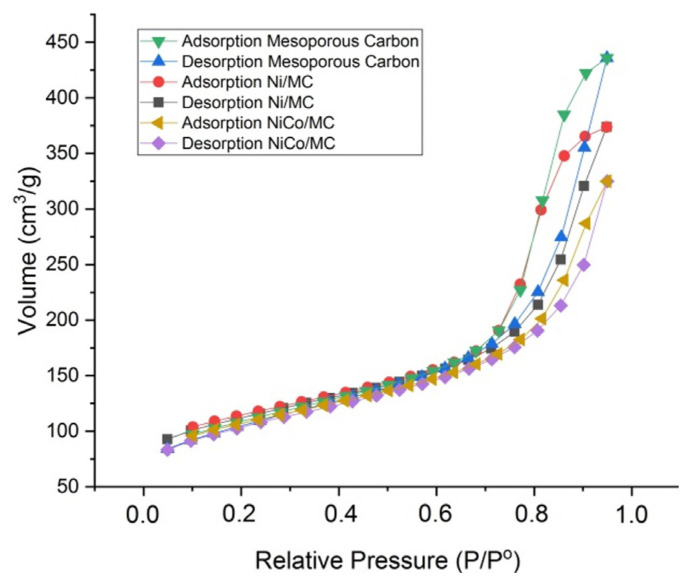
Adsorption-desorption isotherms curve of mesoporous carbon, Ni/MC, and NiCo/MC.

Surface area analysis was used to determine the adsorption-desorption isotherm, surface area and pore distribution of the catalysts. The calculation was carried out using the Brunauer-Emmett-Teller (BET) method to determine the surface area and Barrett-Joyner-Halenda (BJH) method to determine the pore size distribution of the material. Figure 6 shows the isotherm curve of the mesoporous carbon, Ni/MC and NiCo/MC. The mesoporous properties of MC and its derivatives are confirmed by the IV type of adsorption isotherm curve and H-1 hysteresis loop from all curves. The surface properties of all materials are summarized in Table 1. Based on the table, Ni/MC has a larger surface area than mesoporous carbon and NiCo/MC. This is because the nickel was impregnated not only inside the pore but also on the surface of the mesoporous carbon and formed an aggregate outside the pore, thereby increasing the surface area of the Ni/MC catalyst. This is similar to what we have observed in the impregnation of porous carbon with copper [19]. The pore diameter of MC is relatively the same after Ni-impregnation, indicating that the impregnation process did not change the pore structure of the mesoporous carbon. These results support the data obtained from the TEM characterization.

### Catalytic test on selective hydrogenation of diphenylacetylene

**Figure 7:**
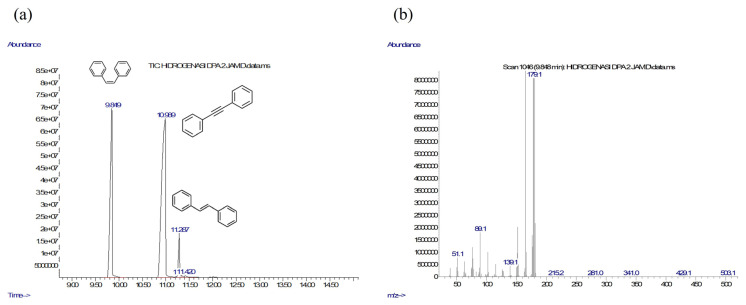
(a) Chromatogram of reaction mixtures (reaction conditions: using NiCo/MC catalyst at 30 °C for 2 h), (b) mass spectrum of cis-stilbene.

**Figure 8:**
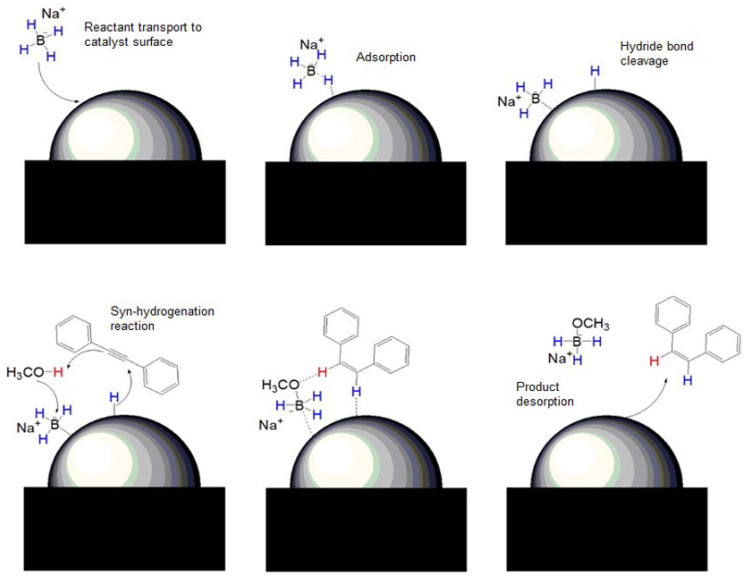
Reaction mechanism for hydrogenation of diphenylacetylene catalyzed by metal (Ni or bimetallic NiCo) supported on MC.

The NiCo/MC and Ni/MC catalyst were tested for their catalytic activity in the hydrogenation reaction of diphenylacetylene with NaBH_4_ to give stilbene as the product. The unreacted diphenylacetylene and product from hydrogenation reaction were analyzed using GC-MS. Typical chromatograms of the reaction mixtures and MS spectra are presented in Figure 7a and Figure 7b, respectively. The chromatogram in Figure 7a shows the presence of unreacted diphenyleacetylene and both *cis*- and *trans*-stilbene as hydrogenated products. Meanwhile, the MS spectrum in Figure 7b shows the fragmentation of the peak that appears at a retention time of 9.848 min which is a *cis*-stilbene compound. Proposed reaction mechanism for the Ni/MC- or NiCo/MC-catalyzed hydrogenation of diphenylacetylene with NaBH_4_ is presented in Figure 8. The hydrogenation was first initiated with hydrogen transport from borohydride to catalyst surface via adsorption. The nucleophilic H on the surface of the catalyst would attack the triple bond in alkyne. Next, methanol as a solvent will bind with boron, and the hydrogen-bond becomes weak, resulting in a second protonation in the triple bond of alkynes to afford stilbene. Hydrogenation reactions using NaBH_4_ and methanol tend to form *cis*-alkene as a major product since methanol is activated by the adsorbed borohydride, thus the hydrogen source from methanol and NaBH_4_ will interact with alkyne via *syn*-hydrogenation manner [4].

**Figure 9:**
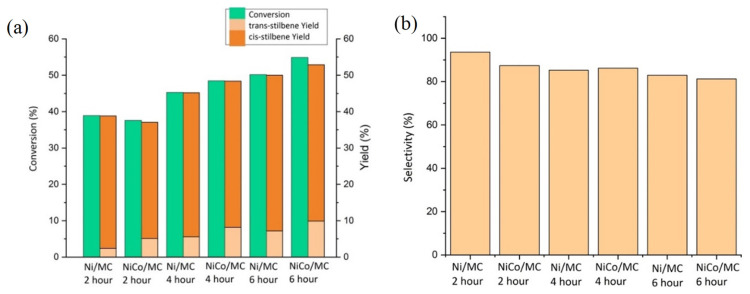
Hydrogenation of diphenylacetylene at room temperature with time variations: (a) diphenylacetylene conversion and yield of products, (b) selectivity of *cis*-stilbene.

First, we investigated the effect of reaction time to diphenylacetylene conversion as well as *cis*-stilbene selectivity for both Ni/MC and NiCo/MC catalysts. At this stage, the reaction was conducted at room temperature. Figure 9a shows that both Ni/MC and NiCo/MC catalysts provide almost the same catalytic activity, in which there is an increase in the conversion of diphenylacetylene with increasing reaction time for both catalysts. Both catalysts also show good selectivity toward the formation of *cis*-stilbene, especially at a relatively short reaction time. However, with increasing reaction time, there was a slight decrease in selectivity towards *cis*-stilbene formation (Figure 9b). It can be understood that as the reaction time goes on, more methanol is activated by the borohydride, thus allowing the addition of the alkyne bond from two different sides to form a *trans*-stilbene product.

**Figure 10:**
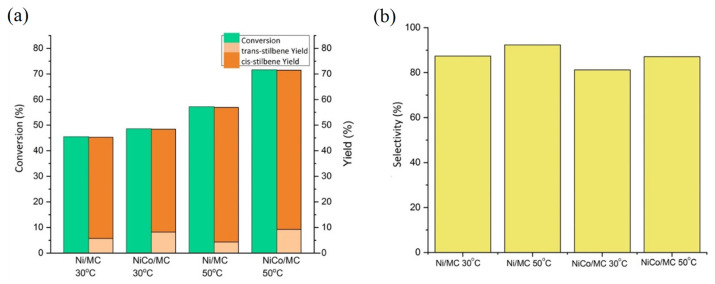
Hydrogenation of diphenylacetylene with variation of temperature reaction: (a) diphenylacetylene conversion and yield of products, (b) selectivity of *cis*-stilbene.

The effect of reaction temperature on the catalytic activity and selectivity of Ni/MC and NiCo/MC was investigated. The reactions were conducted for 4 h. As predicted, there is an increase in the amount of diphenylacetylene converted as reaction temperature raised to 50 °C (Figure 10a). An increment in the selectivity to *cis*-stilbene over the increase of reaction temperature is also observed (Figure 10b). Interestingly, the NiCo/MC gave superior catalytic activity compared to Ni/MC at higher temperature (50 °C) while maintaining selectivity. The optimum conditions were obtained at 50 °C for 4 h with a diphenylacetylene conversion of 71.5% with a selectivity to the *cis*-stilbene formation of 87.1%. The presence of Co in the NiCo bimetal catalyst helped to improve the performance of the catalyst due to the high catalytic activity of cobalt and increased the adsorption capacity of borohydride anions on the surface of the catalyst to form active hydrogen which would interact with the alkyne [24]. These results indicate that the bimetallic NiCo/MC catalyst has promising catalytic activity in the selective hydrogenation of diphenylacetylene. In fact, its catalytic activity can also be compared with the performance of other Ni-based heterogeneous catalysts, or with homogeneous catalysts which generally use high hydrogen gas pressure, as summarized in Table 2.

## 4. Conclusion

Selective hydrogenation of diphenylacetylene over Ni/MC and NiCo/MC catalysts was successfully carried out. The catalysts show good catalytic activity and selectivity for *cis*-stilbene as a major product. The presence of cobalt metal in the bimetallic NiCo/MC significantly improves the catalytic activity at mild temperature while maintaining its good selectivity to *cis*-stilbene.

## Figures and Tables

**Table 1:** Physicochemical properties of Mesoporous Carbon, Ni/MC, and NiCo/MC.

Sample	S_BET_^a^ (m^2^/g)	S_ext_^b^ (m^2^/g)	S_mikro_^b^ (m^2^/g)	V_total_^c^ (cc/g)	V_meso_^d^ (cc/g)	V_micro_^c^ (cc/g)	Pore diameter (nm)
MC	360	219	141	0.5459	0.5051	0.0444	12.81
Ni/MC	376	220	156	0.9018	0.8577	0.0442	13.36
NiCo/MC	353	219	135	0.6939	0.6492	0.0447	12.76

a determined using BET method

b determined using t-plot method

c determined using BJH method

d determined by the difference of Vtotal – Vmicro = Vmeso

**Table 2:** The catalytic activity of NiCo/MC compared to other Ni-based catalysts in the hydrogenation of diphenylacetylene.

Catalyst	Conditions	Results	Ref.
Ni(NO_3_)_2_.6H_2_O, monodentate P-ligand (homogeneous)	120 °C, 30 bar H_2_, CH_3_CN solvent, 15 h	Conversion: 90%–98% Selectivity: 87%–94% (Z)-stilbene, <1% (E)-stilbene	[5]
NiCl_2_ (colloidal Ni NP)	Room temperature, NaBH_4_ (2 equiv.), MeOH solvent	Conversion: 98% Selectivity: 94% (Z)-stilbene, 5% (E)-stilbene	[4]
Bimetallic NiOs_4_ (colloidal nanoparticle)	80 °C, 10 bar H_2_, THF solvent, 6 h	Conversion: ~99% Selectivity: ~83% (Z)-stilbene, ~14% (E)-stilbene, ~3% diphenylethane	[25]
Ni@Y-Zeolite (heterogeneous)	175 °C, 10 bar H_2_, cyclohexane solvent, 6 h	Conversion of : 99% Selectivity: 61% (Z)-stilbene, 39% (E)-stilbene	[26]
Ni-fructose@SiO_2_ (heterogeneous)	110 °C, 10 bar H_2_, CH_3_CN solvent, 15 h	Conversion: >99% Selectivity: 93% (Z)-stilbene, <1% (E)-stilbene, 5% diphenylethane	[27]
NiCo/MC (heterogeneous)	50 °C, NaBH_4_ (2 equiv.), MeOH solvent, 4 h	Conversion: 71.5% Selectivity: 87.1% (Z)-stilbene, 12.9% (E)-stilbene	**This work**

## References

[1] Ritleng V Sirlin C Pfeffer M Ru-, Rh-, and Pd-catalyzed C-C bond formation involving C-H activation and addition on unsaturated substrates: Reactions and mechanistic aspects Chemical Reviews 2002 102 5 1731 1769 10.1021/cr0104330 11996548

[2] Kani I Unver H 2020 Bimetallic Ni(II) complex with carboxylate bridging for homogeneous hydrogenation of alkenes with NaBH_4_ Polyhedron 2020 187 10.1016/j.poly.2020.114649

[3] Özkar S Zahmakiran M Hydrogen generation from hydrolysis of sodium borohydride using Ru(0) nanoclusters as catalyst Journal of Alloys and Compounds 2005 404–406 728 731 10.1016/j.jallcom.2004.10.084

[4] Wen X Shi X Qiao X Wu Z Bai G Ligand-free nickel-catalyzed semihydrogenation of alkynes with sodium borohydride: a highly efficient and selective process for *cis*-alkenes under ambient conditions Chemical Communications 2017 53 39 5372 5375 10.1039/c7cc02140b 28402371

[5] Murugesan K Bheeter CB Linnebank PR Spannenberg A Reek JNH Nickel-Catalyzed Stereodivergent Synthesis of E- and Z-Alkenes by Hydrogenation of Alkynes ChemSusChem 2019 12 14 3363 3369 10.1002/cssc.201900784 30977957 PMC6771912

[6] Alruqi SS Al-Thabaiti SA Khan Z Iron-nickel bimetallic nanoparticles: surfactant assisted synthesis and their catalytic activities Journal of Molecular Liquids 2019 282 448 455 10.1016/j.molliq.2019.03.021

[7] Rodiansono R Dewi AM Husain S Nugroho A Sutomo S Selective Conversion of 2-Methylfuran to 1,4-Pentanediol Catalyzed by Bimetallic Ni-Sn Alloy Bulletin of Chemical Reaction Engineering & Catalysis 2019 14 3 529 541 10.9767/bcrec.14.3.4347.529-541

[8] Seethapathy V Sudarsan P Pandey AK Pandiyan A Kumar THV Synergistic effect of bimetallic Cu:Ni nanoparticles for the efficient catalytic conversion of 4-nitrophenol New Journal of Chemistry 2019 43 3180 3187 10.1039/C8NJ05649H

[9] Khairani NS Abdullah I Krisnandi YK Synthesis and characterization of NiZn/mesoporous carbon as heterogeneous catalyst for carboxylation reaction of acetylene with CO2 AIP Conference Proceedings 2020 2242 1 040039 10.1063/5.0007890

[10] Gallo A Snider JL Sokaras D Nordlund D Kroll T Ni_5_Ga_3_ catalysts for CO_2_ reduction to methanol: Exploring the role of Ga surface oxidation/reduction on catalytic activity Applied Catalysis B: Environmental 2020 267 118369 10.1016/j.apcatb.2019.118369

[11] Pei GX Liu XY Wang A Su Y Li L Selective hydrogenation of acetylene in an ethylene-rich stream over silica supported Ag-Ni bimetallic catalysts Applied Catalysis A: General 2017 545 90 96 10.1016/j.apcata.2017.07.041

[12] Bian Z Das S Wai MH Hongmanorom P Kawi S A Review on Bimetallic Nickel-Based Catalysts for CO_2_ Reforming of Methane ChemPhysChem 2017 18 22 3117 3134 10.1002/cphc.201700529 28710875

[13] Yang L Yu S Peng C Fang X Cheng Z Semihydrogenation of phenylacetylene over nonprecious Ni-based catalysts supported on AlSBA-15 Journal of Catalysis 2019 370 310 320 10.1016/j.jcat.2019.01.012

[14] Ridwan M Suhanda D Aziz I Abdullah I Dehydrogenatıon of hydrazine hydrate using NiCo bimetallic catalyst supported on natural zeolite (ZA), Z-NaY, Z-HY, Al_2_O_3_ and TiO_2_ Rasayan Journal of Chemistry 2021 14 3 1821 1828 10.31788/RJC.2021.1436319

[15] Pal N Bhaumik A Soft templating strategies for the synthesis of mesoporous materials: Inorganic, organic-inorganic hybrid and purely organic solids Advances in Colloid and Interface Science 2013 189–190 21 41 10.1016/j.cis.2012.12.002 23337774

[16] Afriani A Abdullah I Krisnandi YK Synthesıs of NiCl_2_ impregnated mesoporous carbon and its adsorption activity on CO_2_ AIP Conference Proceedings 2021 2349 020048 10.1063/5.0051524

[17] Abdullah I Andriyanti R Nurani DA Krisnandi YK Nickel-phenanthroline complex supported on mesoporous carbon as a catalyst for carboxylation under CO_2_ atmosphere Bulletin of Chemical Reaction Engineering & Catalysis 2021 16 1 111 119 10.9767/bcrec.16.1.9733.111-119

[18] Pamungkas AZ Abdullah I Krisnandi YK Synthesis and characterization of Ni nanoparticles supported on nitrogen-doped mesoporous carbon IOP Conference Series 2019 496 012003 10.1088/1757-899X/496/1/012003

[19] Amalia PN Abdullah I Rahayu DUC Krisnandi YK Synthesis and Characterization of Copper Impregnated Mesoporous Carbon as Heterogeneous Catalyst for Phenylacetylene Carboxylation with Carbon Dioxide Indonesian Journal of Chemistry 2021 21 1 77 87 10.22146/ijc.52778

[20] Zawislak A Choma J Jaroniec M Adsorption and structural properties of soft-templated mesoporous carbons obtained by carbonization at different temperatures and KOH activation Applied Surface Science 2010 256 5187 5190 10.1016/j.apsusc.2009.12.092

[21] Sulistianti I Krisnandi YK Moenandar I Study of CO_2_ adsorption capacity of mesoporous carbon and activated carbon modified by triethylenetetramine (TETA) IOP Conference Series: Materials Science and Engineering 2017 188 012041 10.1088/1757-899X/188/1/012041

[22] He X Xu Y Yao X Zhang C Pu Y Large exchange bias and enhanced coercivity in strongly-coupled Ni/NiO binary nanoparticles RSC Advances 2019 9 52 30195 30206 10.1039/C9RA03242H 35530194 PMC9072138

[23] García T Murillo R Agouram S Dejoz A Lázaro MJ Highly dispersed encapsulated AuPd nanoparticles on ordered mesoporous carbons for the direct synthesis of H_2_O_2_ from molecular oxygen and hydrogen Chemical Communications 2012 48 43 5316 5318 10.1039/c2cc14667c 22513519

[24] Netskina OV Tayban ES Rogov VA Ozerova AM Mukha SA Solid-state NaBH_4_ composites for hydrogen generation: Catalytic activity of nickel and cobalt catalysts International Journal of Hydrogen Energy 2021 46 7 5459 5471 10.1016/j.ijhydene.2020.11.078

[25] Egeberg A Dietrich C Kind C Popescu R Gerthsen D Bimetallic NiIr_4_ and NiOs_4_ alloy nanoparticles and their catalytic performance in hydrogenation reactions ChemCatChem 2017 9 18 3534 3543 10.1002/cctc.201700168

[26] Deng X Bai R Chai Y Hu Z Guan N Homogeneous-like alkyne selective hydrogenation catalyzed by cationic nickel confined in zeolite Chinese Chemical Society Chemistry 2021 3 1101 1114 10.31635/ccschem.021.202100820

[27] Murugesan K Alshammari AS Sohail M Beller M Jagadeesh RV Monodısperse nickel-nanoparticles for stereo- and chemoselective hydrogenation of alkynes to alkenes Journal of Catalysis 2019 370 372 377 10.1016/j.jcat.2018.12.018

